# Demonstration of Efficient Ultrathin Side-Emitting InGaN/GaN Flip-Chip Light-Emitting Diodes by Double Side Reflectors

**DOI:** 10.3390/nano12081342

**Published:** 2022-04-13

**Authors:** Tae Kyoung Kim, Abu Bashar Mohammad Hamidul Islam, Yu-Jung Cha, Seung Hyun Oh, Joon Seop Kwak

**Affiliations:** 1 Department of Energy Engineering, Korea Institute of Energy Technology, Naju-si 58330, Jeollanam-do, Korea; tkkim@kentech.ac.kr (T.K.K.); abmhis@kth.se (A.B.M.H.I.); yjcha@kentech.ac.kr (Y.-J.C.); 2 Lumens Co., Ltd., Yongin 446901, Gyeonggi-do, Korea; oh.dave@lumens.co.kr

**Keywords:** light-emitting diodes, ultrathin side-emitting, ZnO, nanorods, light output power, light extraction efficiency

## Abstract

This work proposes an InGaN/GaN multiple-quantum-well flip-chip blue ultrathin side-emitting (USE) light-emitting diode (LED) and describes the sidewall light emission characteristics for the application of backlight units in display technology. The USE-LEDs are fabricated with top (ITO/distributed Bragg reflector) and bottom (Ag) mirrors that cause light emission from the four sidewalls in a lateral direction. The effect of light output power (LOP) on lateral direction is consistently investigated for improving the optoelectronic performances of USE-LEDs. Initially, the reference USE-LED suffers from very low LOP because of poor light extraction efficiency (LEE). Therefore, the LEE is improved by fabricating ZnO nanorods at each sidewall through hydrothermal method. The effects of ZnO nanorod lengths and diameters on LOP are systematically investigated for optimizing the dimensions of ZnO nanorods. The optimized ZnO nanorods improve the LEE of USE-LED, which thus results in increasing the LOP > 80% compared to the reference LED. In addition, the light-tools simulator is also used for elucidating the increase in LEE of ZnO nanorods USE-LED.

## 1. Introduction

Nitride-based multiple-quantum-well (MQW) light-emitting diodes (LEDs) have been applied for different kinds of commercial applications such as full-color displays, traffic displays, indoor and outdoor lighting, automobiles, and backlight units of liquid crystal displays (LCDs) because they are more reliable, efficient, cost effective, and have longer life spans [1,2,3,4,5]. These applications require high light output power (LOP). Generally, LOP decreases on account of low internal quantum efficiency (IQE) and light extraction efficiency (LEE) caused by the high defect density and low light escaping probability since 80% of the emitted photons in a conventional lateral LED structure are totally reflected from the interface of GaN-based epitaxial layer (*n* = 2.5) and air (*n* = 1), respectively. Various research has been proposed for improving the device IQE [6,7,8]. On the other hand, the patterned sapphire substrate (PSS) not only enhances the light-escaping probability but also improves the crystal quality by reducing the threading dislocations [9]. The LEE of GaN-based LEDs can also increase by designing bottom emission LEDs, especially vertical [10] and flip-chip [11,12,13] LEDs because of the smaller difference in refractive indexes between sapphire (*n* = 1.78) and air (*n* = 1).

Despite the limitation of LOP, the GaN-based LEDs are widely used in LCD technology where a single LED works like a pointed light source that is then converted into a sheet light source for an LCD backlight unit [14]. In addition, the solid-state based LCD technology has gradually become mature because of extensive research in material, device innovation, and finally the development of advanced manufacturing technologies. Most of the crucial issues of LCDs, namely, the power consumption, the viewing angle, the contrast ratio, the response time, and the color gamut, have been overcome to an acceptable level [14,15,16,17,18]. Therefore, LCDs are the dominant display technology for various kinds of applications from projectors, televisions, monitors, and tablets to smartphones [19,20]. Currently, organic LED (OLED) displays are a rapidly growing technology and are trying to capture the applications and market of LCDs [21,22], which therefore results in causing a big challenge for the existence of LCDs in the near future. Both LCD and OLED display technologies have their pros and cons [23,24]. In order to generate images, the LCD requires an LED backlight unit [14,16,17,18] for emitting light through a matrix, whereas OLED display does not require any backlight because of a self-emissive property. The most critical issues of OLED displays are color reliability and stability because of its inorganic nature [21,22,24]. In addition, the OLED-based flat panel displays (FPDs) are much thinner than those of LCD-based FPDs. The further development of thin to thinner OLED displays will reduce the future possible uses of LCD displays. The thickness of LCD-based FPD increases because of its use of thick lenses on the LED backlight for propagating radiation pattern to the lateral direction, and the air gap between backlight and diffuser sheet, which are inevitable issues in LCD technology. The thick lens from the top of an LED backlight can be removed; somehow, the light directly comes out from an LED in lateral direction without any lens. This can be possible as the light comes out from the four sidewalls of an LED. However, there is no progressive research or report on the optoelectronic performances of edge emitters. To address this issue, the flip-chip ultrathin side-emitting (USE) LEDs will be a potential candidate for next generation LED backlight and also attract for other novel applications.

In this research work, we fabricate InGaN/GaN MQWs flip-chip blue USE-LEDs with top and bottom mirrors to investigate the optoelectronics characteristics for the application of backlight unit. For increasing the LOP of a USE-LED, the ZnO nanorods are fabricated on each sidewall. Therefore, the dependency of LOP on the ZnO nanorods length and diameter is systematically investigated for optimizing the dimension of ZnO nanorods. The optoelectronic characteristics of optimized ZnO nanorods-based USE-LED are also compared with the Reference LED. The light-tools simulator is also used for elucidating the increase in LOP of ZnO nanorods-based USE-LED.

## 2. Materials and Methods

### Device Structure and Experiments

The similar epitaxial structure InGaN/GaN-base blue LEDs are grown on c-plane PSS by metal-organic chemical vapor deposition (MOCVD). The basic LED epitaxial structure consists of a 40 nm thick undoped GaN buffer layer, a 5.5 μm thick Si doped n-GaN layer (5 × 10^18^ cm^−3^), and a 120 nm thick InGaN/GaN superlattice, which were consecutively grown on the substrate. The active layer composed of five pairs of 2.3 nm thick In_0.18_Ga_0.82_N wells separated by the 5 nm thick GaN barriers. A 40 nm thick Mg-doped p-Al_0.2_Ga_0.8_N electron-blocking layer, and finally a 100 nm thick Mg-doped p-GaN (5 × 10^17^ cm^−3^) layer were successfully grown on the active layer for finishing the LED structure.

**Figure 1:**
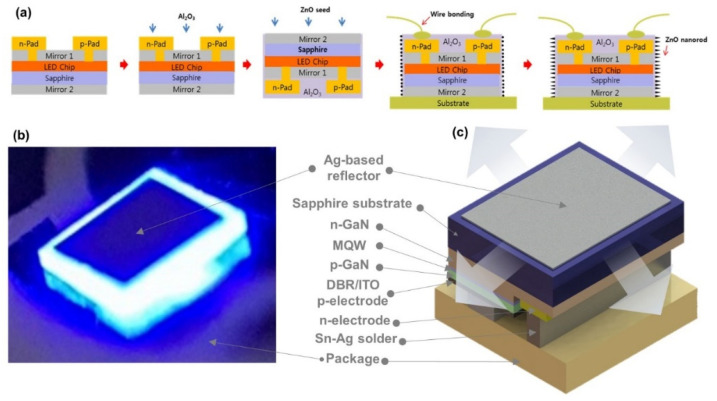
(**a**) The schematic fabrication process flowchart of a USE-LED. (**b**) Light emission from the sidewalls. (**c**) Detailed description of a flip-chip USE-LED.

After epitaxial growth, thermal annealing at 725 °C was performed in a nitrogen atmosphere for 15 min to obtain the p-type conductivity of Mg-doped p-GaN layer. The wafer was then put into deionized (DI) water followed by buffered oxide etchant (BOE) for surface wetting, and removing the surface oxide, respectively. The GaN surface was cleaned with acetone, isopropyl-alcohol, and DI water for 3 min at each step. A standard photolithographic technique was used to define the LED patterns on the p-GaN layer. For exposing the n-GaN layer until a depth of 1 μm, the mesa etching was performed by inductive-coupled-plasma (ICP) with an Ar/Cl_2_ gas mixture. These samples were sequentially cleaned, rinsed, and dried with hydrofluoric acid (HF) solution (HF:DI = 1:1) for 5 s, DI water, and N_2_, respectively. A 200 nm thick ITO contact layer was deposited on the p-GaN surface by an electron beam evaporator (EBE), followed by a Cr/Al/Ni/Au/Cr (30/100/200/200/30 nm) electrode that was deposited on the ITO and n-GaN layers by an EBE. Next, a distributed Bragg reflector (DBR) structure, defined as “Mirror 1”, consisted of 20 pairs of SiO_2_ and TiO_2_ layers that were deposited using radio-frequency (RF) magnetron reactive sputter. At a normal incident of light, the DBR has reflectance >95% in the wavelength ranges from 400 to 700 nm as reported in our previous work [25]. The current injected into n-type and p-type electrodes through the pad layer. Usually, the pad layer was connected with the electrodes via-hole structures that were fabricated on the DBR by photolithography patterning and then reactive ion etching (RIE) with a CF_4_/O_2_ gas mixture as described in Ref. [25]. A total of 28 holes were fabricated on the DBR according to the direction of p-type (16 holes) and n-type (12 holes) electrodes. Instead of using a single hole, a number of holes were fabricated for connecting each electrode, which will reduce the current crowding effect and improve the heat dissipation property from the LED device [25]. To make the contacts with p- and n-type electrodes, contact pads Cr/Al/Ni/Au (0.3/2/0.4/0.2 μm) were selectively deposited on the DBR and holes by an EBE. For insulation, a 100 nm thick Al_2_O_3_ layer was deposited on the pads and the DBR by atomic layer deposition. The highly reflective second mirror, defined as “Mirror 2”, was also fabricated on the polished sapphire substrate by depositing a 300 nm thick Ag film through the RF magnetron sputter. For improving LOP, the ZnO nanorods were fabricated at each sidewall of a USE-LED. First, a 100 nm thick ZnO seed layer was deposited by sputter, and then nanorods were grown on the seed layer by hydrothermal method. The detailed growth conditions were described in our previous work [26]. Finally, a flip-chip 700 × 700 μm^2^ LED device was bonded on the package for measuring the various optoelectronic performances at room temperature. The simplified fabrication steps of a flip-chip USE-LED are described in Figure 1a. The fabricated Mirror 1 and Mirror 2 prevent the emitted photons from passing through both top and bottom faces. Thus, the emitted photons only pass in a lateral direction through the sidewalls, i.e., working as an edge emitting diode [27] as shown in Figure 1b, and the detailed description of a flip-chip USE-LED is presented in Figure 1c.

The current-voltage (*I-V*) characteristics of USE-LED devices were measured by a Keithley 2602B (Keithley Instruments, Cleveland, OH, USA) source meter with a voltage sweep rate of 0.35 V·s^−1^. A Si p-i-n photodiode (S2281-04, Hamamatsu Photonics, Hamamatsu, Japan) was used for measuring the electroluminescent (EL) intensity. A high-resolution charge-coupled-device (CCD) camera (ISH300, Tucsen, Gaishan, China) was used for recording the two-dimensional EL images. A fiber-optic spectrometer (AvaSpec-2048, Avantes, Apeldoorn, Netherlands) was used to record the peak emission wavelength of LED devices. The real LOP vs. current (*L-I*) was measured through an integrating sphere system connected with a spectrometer (CAS 140 CT, Instrument Systems, Munich, Germany) made by Instrument Systems. An ultrahigh resolution Schottky field emission SEM (JEOL JSM-7610F Plus, Pleasanton, CA, USA) was used for investigating the morphology of ZnO nanorods.

## 3. Results and Discussion

**Figure 2:**
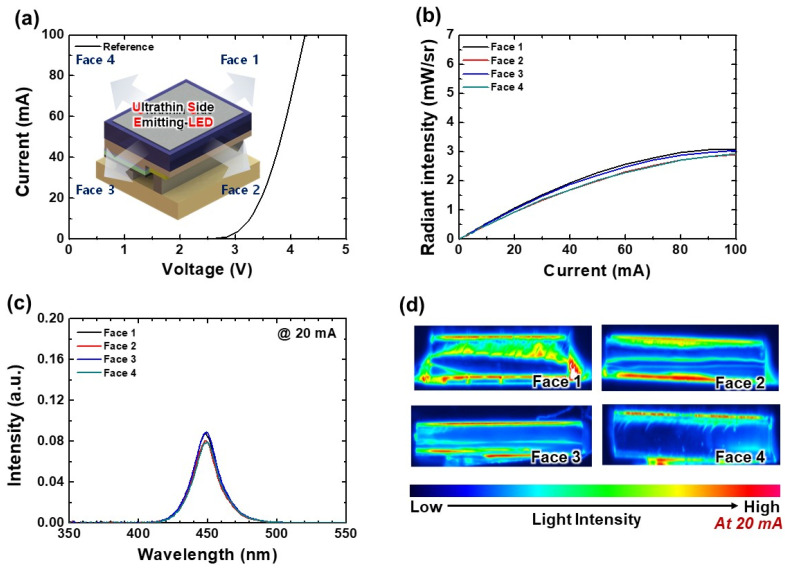
(**a**) *I-V* characteristics of a USE-LED (Reference sample). Inset shows the schematic diagram for light emitting from its 4 Faces. (**b**) LOP characteristics, (**c**) EL spectra at 20 mA, and (**d**) light intensity distribution over each Face (or, sidewall) of a USE-LED.

Figure 2 shows the basic optoelectronic characteristics of a USE-LED, which is defined as a “Reference LED” sample. The measured *I-V* plot is presented in Figure 2a. The inset describes that the light emits from the 4 Faces (or, sidewalls) of a flip-chip USE-LED. The LOP characteristics of 4 Faces are shown in Figure 2b. All Faces have about similar LOP characteristics. At 100 mA, the radiant intensities of LOPs at Face 1, 2, 3, and 4 are about 3.0, 2.9, 3.0, and 2.9 mW/st, respectively. The total LOP of 4 Faces is about 12 mW/st at 100 mA. At 20 mA, the measured EL spectra of 4 Faces are described in Figure 2c. The EL spectra curves present that the peak wavelength of each Face is about 450 nm. At 20 mA, the light intensity distribution over each sidewall area is also measured in order to elucidate the low LOP of a USE-LED as presented in Figure 2d. It is observed that the light intensity distribution over the sidewall area of each Face is not uniform, which is due to the poor LEE. Therefore, the LEE of a USE-LED needs to increase for its extensive application as a backlight unit in LCD technology.

**Figure 3:**
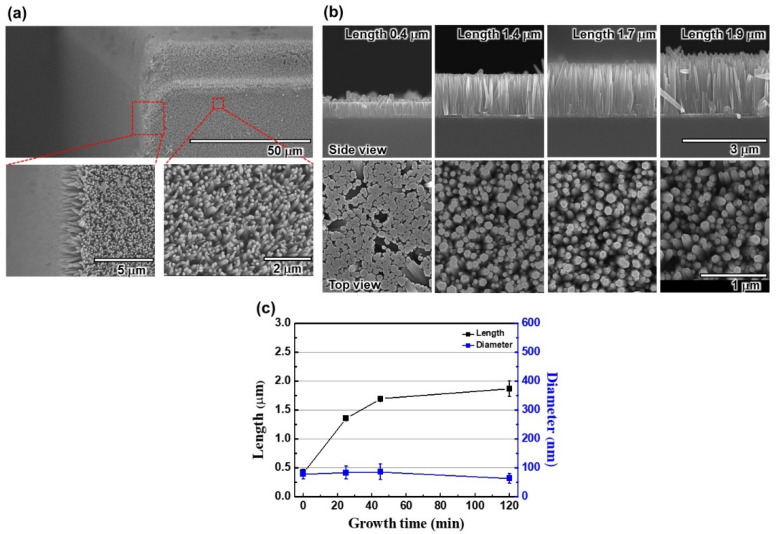
(**a**) SEM characteristics of fabricated ZnO nanorods on the sidewalls of a USE-LED. (**b**) SEM characteristics of various ZnO nanorods’ lengths and diameters for side and top views, respectively. (**c**) Variation of ZnO nanorods’ lengths with the growth times.

The LEE of a USE-LED can be increased by fabricating ZnO nanorods at each sidewall as described in refs. [26,28]. The ZnO is used for fabricating nanorods because of the reduction of the Fresnel reflection at the interface between the MQWs and ZnO nanorods by reducing the difference in refractive indices between the MQWs and ZnO nanorods. It indicates that more photons could be confined in nanorods and then propagated through the nanorods, where a nanorod acts as a waveguide owing to high refractive index nanorods covered by the low refractive index air. The morphology of fabricated ZnO nanorods on the sidewalls of a USE-LED is shown in Figure 3a. It is important to note that the nanorods are fabricated uniformly on the sidewalls as seen in the SEM characteristics. Four different kinds of similar epitaxial structure USE-LEDs were fabricated by varying the ZnO nanorods’ lengths from 0.4 to 1.9 μm. During deposition, the chamber pressure, the stirrer revolution rate, and the growth temperature were 50 kg/cm^2^, 2000 RPM, and 90 °C, respectively. At 60 mmol solution concentration, the lengths of nanorods were varied by increasing the deposition time [26]. Figure 3b shows the SEM characteristics of fabricated ZnO nanorods. The variation of length and diameter vs. growth time plots are described in Figure 3c. We observed that the length of the fabricated nanorods increases with increasing the growth time; however, the diameter of nanorods is about equal. At a similar growth condition, the nanorods lengths are 0.4, 1.4, 1.7, and 1.9 μm for the growth times 0, 25, 60, and 120 min, respectively. More significantly, the average diameter of ZnO nanorods is about 78 nm.

**Figure 4:**
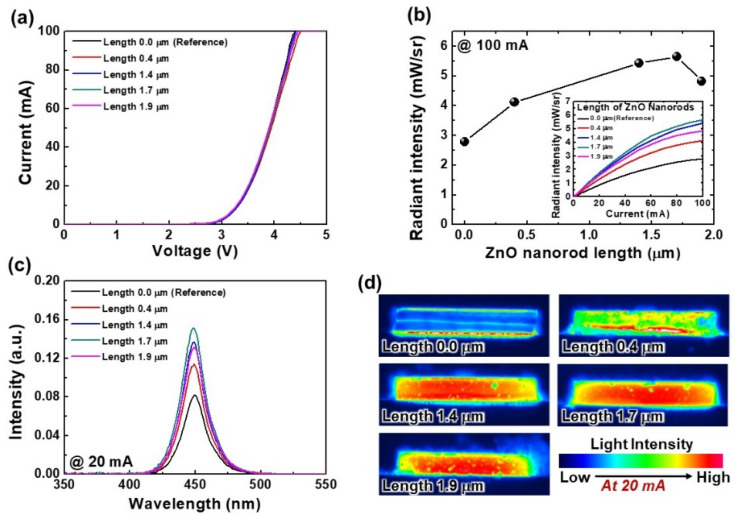
(**a**) *I-V* characteristics of reference and various lengths of ZnO nanorods USE-LEDs. (**b**) Change in peak amplitude of LOP with the variation of ZnO nanorods length at 100 mA. Inset shows the current vs. LOP characteristics of LED devices. (**c**) EL spectra, and (**d**) EL intensity distribution over the sidewall area at 20 mA for without and with various lengths of ZnO nanorods USE-LEDs.

Figure 4 shows the optoelectronic characteristics of USE-LEDs for without and with various lengths of ZnO nanorods at similar diameter (about 78 nm see Figure 3). The measured *I-V* plots are shown in Figure 3a. More interestingly, the *I-V* curve demonstrates that there is no observable difference between without and with ZnO nanorods USE-LEDs, since ZnO nanorods do not have any effect on the current injection and recombination in the active region. At 100 mA, the LOPs of USE-LEDs are about 2.8, 4.1, 5.4, 5.7, and 4.8 mW/sr for nanorods lengths 0, 0.4, 1.4, 1.7, and 1.9 μm, respectively, as shown in Figure 4b. The LOP increases with increasing the length of nanorods from 0 to 1.7 μm. After that, the LOP decreases for further increase in nanorod length. The USE-LEDs with ZnO nanorods have higher LOP than the without ZnO nanorods USE-LED. This might suggest that there is an increase in LEE caused by the fabrication of ZnO nanorods because presented USE-LEDs have similar diffusion current and carrier recombination at about same current injection (see *I-V* curves of Figure 4). The inset shows the radiant intensity of LOP vs. current plots. At 20 mA, the effect of nanorods lengths on the peak amplitude of EL spectra is presented in Figure 4c. The EL curves show that the presented USE-LEDs have peak amplitude at about 450 nm; however, as described earlier, the peak amplitude of EL spectra is also increased similar to the LOP. To understand the origin of increase in LOP, the light intensity distribution over the sidewall area is also measured as described in Figure 4d. The light distribution images consistently reveal that the light intensity distribution over the sidewall area also increases with the nanorod’s length, since ZnO nanorods only have effect on the LEE of the LED devices [26]. The USE-LED with nanorods with a length of 1.7 μm has more uniform light intensity distribution than other LED devices. The improved uniform light intensity distribution indicates that the LEE increases with nanorod length. As a result, the LOP of a USE-LED increases in a lateral direction. The decrease in LOP of a 1.9 μm long nanorod USE-LED is due to the absorption of light caused by the longer nanorods.

**Figure 5:**
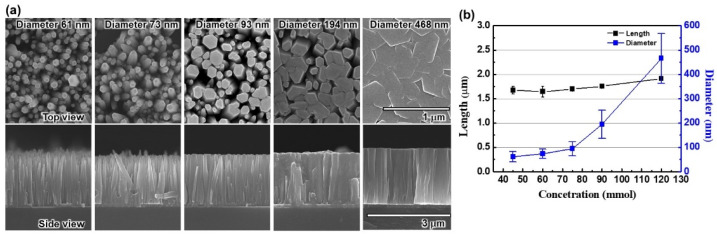
(**a**) SEM characteristics of various diameter- and length-based ZnO nanorods for side and top views, respectively. (**b**) Variation of ZnO nanorod lengths and diameters with the solution concentration.

The SEM images for different diameter ZnO nanorods are described in Figure 5a. The growth conditions of different diameter nanorods were similar to the growth conditions of different length nanorods, except solution concentration and growth time. Five similar epitaxial structure USE-LEDs were fabricated by varying the diameters from 61 nm to 486 nm. At about similar growth conditions, the nanorod diameters are 61, 73, 93, 194, and 486 nm for the solution concentration 45, 60, 75, 90, and 120 mmol, and growth time 180, 60, 45, 32, and 120 min, respectively, as shown in Figure 5b. It is important to note that the length of nanorods is about 1.75 μm.

**Figure 6:**
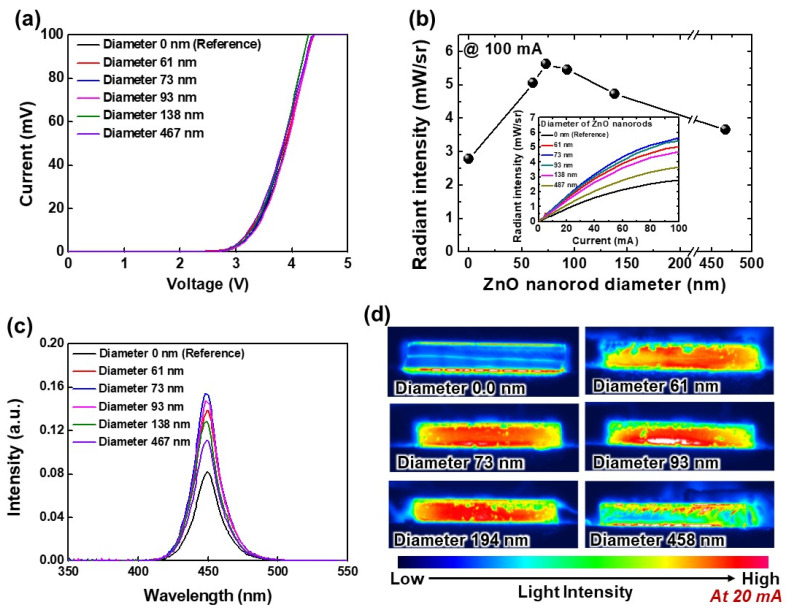
(**a**) *I-V* characteristics of reference and various diameters nanorod-base USE-LEDs. (**b**) Change in LOP with the variation of ZnO nanorod diameters at 100 mA. Inset shows the current vs. LOP characteristics of USE-LED devices. (**c**) EL spectra, and (**d**) EL intensity distribution over the sidewall area at 20 mA for without and with various diameters nanorod-based USE-LEDs.

Figure 6 shows the optoelectronic characteristics of USE-LEDs for various diameters of ZnO nanorods at similar length (about 1.75 μm see Figure 5). The measured *I-V* plots of without and with ZnO nanorods USE-LEDs are shown in Figure 6a. The *I-V* curves reveal that there is no observable difference between reference and with ZnO nanorods USE-LEDs as described earlier. At 100 mA, the radiant intensities of LOPs of USE-LEDs are about 2.8, 5.1, 5.6, 5.4, 4.7, and 3.7 mW/sr for nanorod diameters about 0, 61, 73, 93, 138, and 467 nm, respectively, as shown in Figure 6b. The LOP increases with increasing nanorod diameters from 0 nm to 73 nm, and then, the LOP decreases for further increase in nanorod diameters. At diameters from 61 nm to 73 nm, the nanorod distribution density is about uniform; otherwise, the nanorod distribution density increases with nanorod diameter as seen in Figure 5a. It is observed that the surface of nanorod diameter 467 nm looks like a film surface because big-sized nanorods are almost attached together. Usually, a waveguide supports a number of modes for specific light propagation directions, which depends on the thickness of waveguide [28]. Therefore, the photon confinement changes with the nanorod diameters. In addition, the confined photons travel more in horizontal direction of the nanorod as the diameters of nanorods increase. This also causes the absorption of light. Therefore, a nanorod diameter ≥ 138 nm has less LOP. On the other hand, nanorod diameters from 50 to 100 nm support a number of waveguide modes compared to other nanorod diameters. Consequently, many photons are confined and then propagated through the surface of nanorods, which therefore results in improving the radiant intensity of LOP. It is also observed that the variation of nanorod diameters changes the nanorod distribution density as seen in Figure 5a. This distribution density is also an important factor for affecting the LEE by confining the light. The USE-LEDs with ZnO nanorods have higher LOP compared to the reference LED. This might also suggest that the LEE also increases as the nanorod diameter changes. The inset shows the LOP vs. current plots for various diameter ZnO nanorods. At 20 mA, the effect of nanorod diameters on the EL spectra is presented in Figure 6c. The EL curves show that the fabricated USE-LEDs have peak amplitude at about 450 nm; however, the peak amplitude of EL spectra changes with nanorod diameter, which might be the effect of nanorod diameter on the LEE. For understanding the origin of increased LOP and EL peak amplitude, the light intensity distribution over the sidewall area is also measured with increasing nanorod diameters as shown in Figure 6d. The light intensity distribution images reveal that the light intensity distribution over the sidewall area increases as the nanorod diameter increases from 0 nm to 73 nm, and then the light intensity distribution decreases for further increases in nanorod diameters. As described earlier, the increased light intensity distribution is due to the improved LEE, and the decreased light intensity distribution is owing to absorption of light by the big-sized nanorod diameter. More significantly, the 73 nm wider diameter nanorod USE-LED has uniform light intensity.

**Figure 7:**
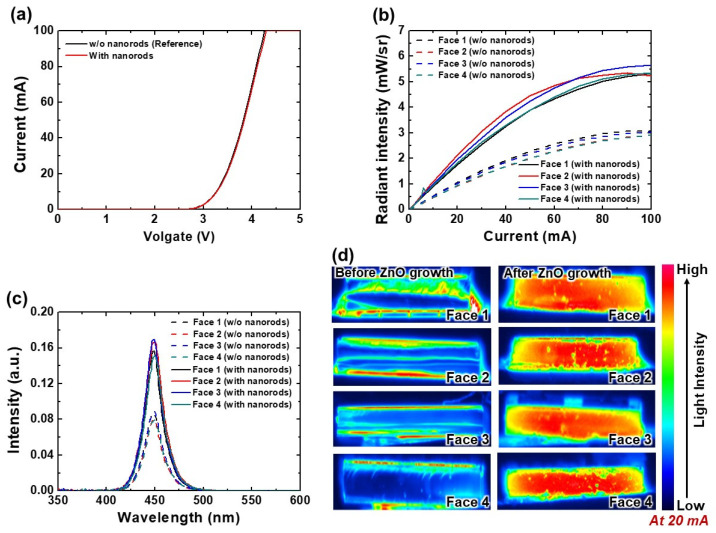
(**a**) *I-V* and, (**b**) LOP characteristics of reference and optimized ZnO nanorods USE-LEDs. (**c**) Peak amplitude of EL characteristics, and (**d**) EL intensity distribution over the sidewall area at 20 mA for reference and optimized ZnO nanorods USE-LEDs.

The optimum length and diameter of ZnO nanorods are consistently found by investigating the effects of lengths and diameters on optoelectronic characteristics (see Figure 4 and Figure 6). Thus, a USE-LED with optimized nanorod lengths (1.7 μm) and diameters (73 nm) is also fabricated, and then compared with the reference LED as shown in Figure 7. The measured *I-V* plots of reference and USE-LED with optimized ZnO nanorods are described in Figure 7a. We observe that the *I-V* curves have no noticeable difference as described earlier. The optimized USE-LED has higher LOP than the reference LED as found in Figure 7b, and each Face has about similar LOP for each LED device. At 100 mA, the radiant intensities of LOPs at Faces 1, 2, 3, and 4 are 5.32, 5.24, 5.67, and 5.35 mW/sr, and 3.0, 2.9, 3.0, and 2.9 mW/sr for optimized and reference USE-LEDs, respectively. More significantly, the LOP of optimized USE-LED increases more than 80% compared to the reference LED. At each Face, the EL spectra of reference and optimized USE-LEDs are plotted in Figure 7c. The EL spectra curves reveal that the peak amplitudes of EL spectra at each Face are about 450 nm. To understand the origin of increase in LOP and EL intensity, the light intensity distribution over the sidewall area is also measured as presented in Figure 7d. The light distribution images reveal that the light intensity distribution at each Face of optimized USE-LED is uniform and higher than the reference device. More significantly, it represents that the optimized ZnO nanorods uniformly improve the LEE at each Face of a USE-LED.

**Figure 8:**
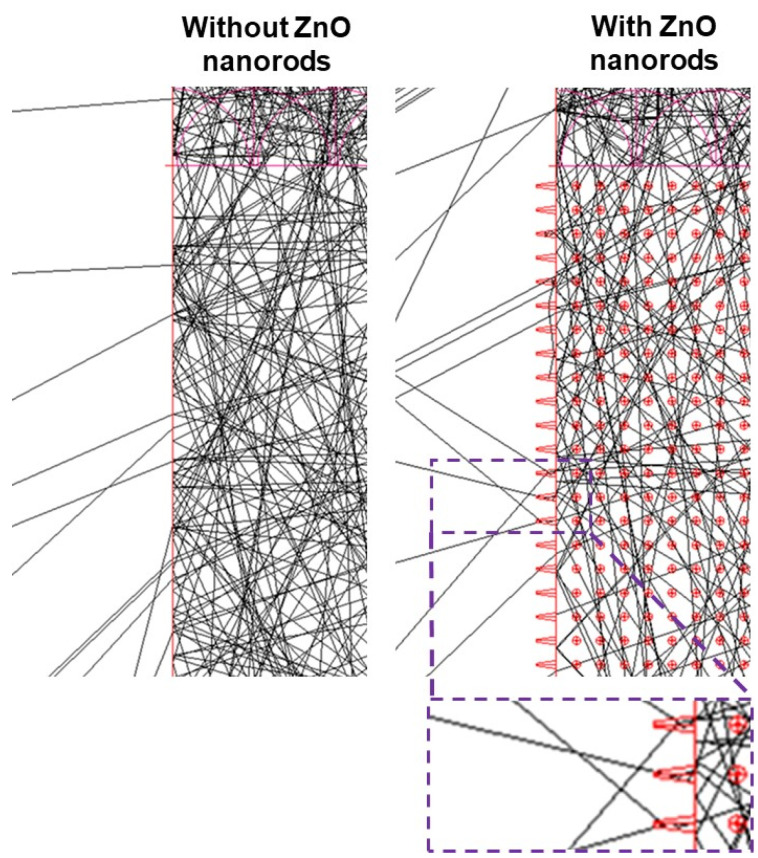
Effect of without/with ZnO nanorods on the LEE for USE-LEDs.

The light-tools simulator is also used to investigate the effect of ZnO nanorods on LEE as shown in Figure 8. The refractive indexes of air, ZnO, and Al_2_O_3_ are 1, 2.0, and 1.7, respectively. The length and diameter of nanorods were 1.7 μm and 77.8 nm, respectively. The ray-tracing simulation was followed by a method as described in ref [29], where the above mention device structure was used for this simulation. The reference USE-LED suffers from internal reflection of light because of Fresnel reflection that causes the LEE to decrease. On the other hand, the USE-LED with ZnO nanorods has less internal reflection of light than the reference LED because nanorods improve the LEE that causes the LOP to increase. This is also consistent with the experimentally obtained optoelectronic characteristics as described in Figure 7. The lateral LOP of optimized USE-LED increases since the photons are guided through the ZnO nanorods layer. In LCD, the LEDs are used as back-light point sources, the radiation patterns of which should be in a lateral direction for propagating light properly to the diffuser sheet. Usually, a thick lens is used on the LED for guiding the radiation pattern to the lateral direction. In USE-LED, the radiation pattern can easily propagate to the lateral direction without any lens. Therefore, the USE-LED will be a suitable candidate as a backlight source in LCD technology for reducing the thickness of LCD since it will eliminate the lens from the LEDs.

## 4. Conclusions

In summary, we have significantly and interactively investigated the effect of LOP on lateral direction of InGaN/GaN MQW flip-chip blue USE-LEDs. The presented USE-LED emits light from its four sidewalls in a lateral direction because LED devices consist of highly reflective top (ITO/distributed Bragg reflector) and bottom (Ag) mirrors. The *I-V* curves reveal that the reference and optimized nanorods-based USE-LEDs have no observable difference because of having similar diffusion current and carrier recombination. However, the optimized nanorods-based USE-LED has higher LOP, peak amplitude of EL spectra, and uniform EL distribution at each Face compared to the reference LED. The LOP of a USE-LED is increased by optimizing the dimensions of ZnO nanorods at each sidewall since ZnO nanorods improve the LEE. In addition, the EL spectra consistently describe that the USE-LED has similar peak wavelength at about 450 nm for each Face. It is clearly found from the light-tools simulation that the LEE of nanorods-based USE-LED increases in a lateral direction because of a decrease in internal reflection of light compared to reference LED, which is also consistent with the experimental characteristics. The lateral LOP of optimized nanorods-based USE-LED increases more than 80% compared to the reference LED. A thick lens on the top of an LED backlight unit does not require to propagate radiation pattern in the lateral direction. Therefore, we anticipate that the USE-LED will be a promising next generation backlight unit for making next generation LCD display.

## Data Availability

The data are available upon reasonable request from the corresponding author.
